# RNA Captor: A Tool for RNA Characterization

**DOI:** 10.1371/journal.pone.0018445

**Published:** 2011-04-13

**Authors:** Christian Clepet

**Affiliations:** URGV Plant Genomics, INRA UMR1165 UEVE/CNRS ERL 8196, Evry, France; University of Cambridge, United Kingdom

## Abstract

**Background:**

In the genome era, characterizing the structure and the function of RNA molecules remains a major challenge. Alternative transcripts and non-protein-coding genes are poorly recognized by the current genome-annotation algorithms and efficient tools are needed to isolate the less-abundant or stable RNAs.

**Results:**

A universal RNA-tagging method using the T4 RNA ligase 2 and special adapters is reported. Based on this system, protocols for RACE PCR and full-length cDNA library construction have been developed. The RNA tagging conditions were thoroughly optimized and compared to previous methods by using a biochemical oligonucleotide tagging assay and RACE PCRs on a range of transcripts. In addition, two large-scale full-length cDNA inventories relying on this method are presented.

**Conclusion:**

The RNA Captor is a straightforward and accessible protocol. The sensitivity of this approach was shown to be higher compared to previous methods, and applicable on messenger RNAs, non-protein-coding RNAs, transcription-start sites and microRNA-directed cleavage sites of transcripts. This strategy could also be used to study other classes of RNA and in deep sequencing experiments.

## Introduction

The RNA world is appearing more and more complex and deciphering the growing list of RNA species, isoforms and byproducts is a major challenge in biology [1]. Genome annotation algorithms are still limited and full-length cDNAs or RACE PCR remain essential for studying the structure and function of the genes. These approaches are widely used for single gene as well as large-scale genomic programs (*e.g.* [2], [3]).

The main limitation full-length cDNA or 5′ RACE methods try to resolve is binding a known sequence at the cap site, so as to prime second-strand polymerization of the cDNA. In some methods, cap-dependent tagging is used as a way of selecting for complete cDNAs; in other protocols the tag is added on cDNAs previously enriched for molecules extending to the 5′cap [4], [5]. Enzymatic tagging can be performed on cDNA or RNA, with as diverse activities as terminal transferase [6], T4 RNA ligase 1 (Rnl1) (*e.g.* [7]–[10]) or reverse transcriptase [11]. More recently we proposed an RNA-tagging solution using the T4 DNA ligase (Dnl) and a special adapter producing double-strand structure at the junction with the RNA [12]. In the framework of the grapevine genome program, 5 full-length cDNA libraries were constructed with this method and 84,000 clones were sequenced. At least 81.6% of the 5′ reads matching to *Arabidopsis* proteins are large enough to span the start codon in this collection used for grapevine genome annotation [13].

The T4 RNA ligase 2 (Rnl2) shows optimal activity on double-strand nucleic acids [14], though it is also used for adding a 3′-adapter by single-strand ligation in small-RNA cloning with the Rnl2(1–249) truncated enzyme producing reduced ligation side products [15], [16]. Based on the full-Rnl2 enzyme and our previous strategy [12], here is shown the development of an effective 5′-RNA-tagging system and its application to RACE PCRs and full-length cDNA library construction. This new method has been used in two large-scale transcript-inventory programs in grapevine and melon. The present report focuses on the implementation and validation of the protocol; its sensitivity as compared to previous methods is shown by RACE PCR on a range of transcripts.

## Results

### High-performance RNA tagging using Rnl2 for transcript analysis

**Figure 1:**
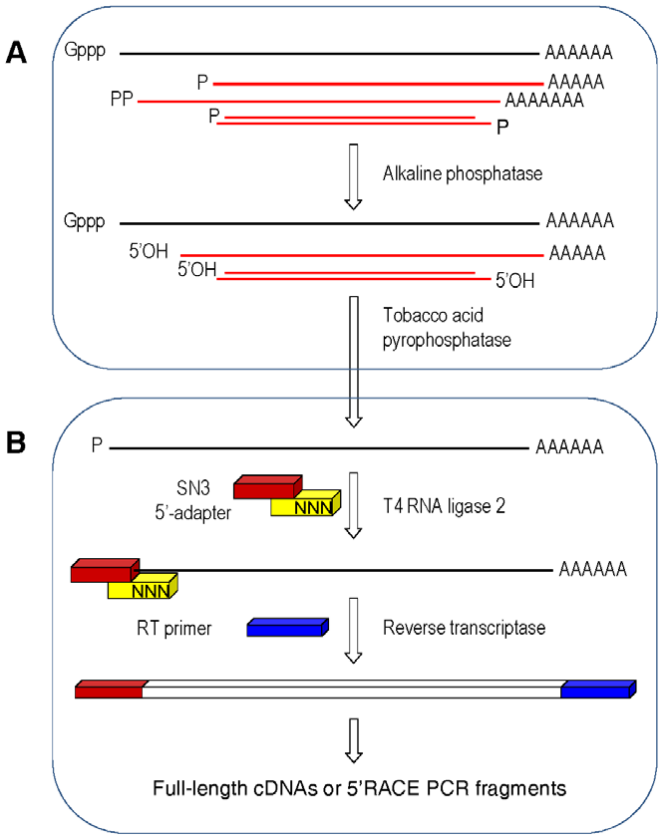
The RNA Captor protocol workflow. Production of cDNAs from capped (**A**) or uncapped (**B**) RNAs. (**A**) Uncapped nucleic acids are inactivated by alkaline phosphatase and the transcripts are decapped by tobacco acid pyrophosphatase. (**B**) Rnl2 is used to add an oligoribonucleotide to the RNA 5′-phosphate by means of a 5′-adapter generating double-stranded structure. The S adapter upper fragment bears a *Sfi*1 site and a ligase-acceptor site. The N3 lower strand has a 5′-random-sticky end to pair with RNA 5′-ends and a 3′-amine to block unwanted ligations. The cDNAs can be reverse transcribed with oligo-dT (SfidTr) or random primers and used for RACE PCRs, full-length cDNA library construction or deep sequencing. P: 5′mono-phosphate. PP: 5′di- or tri-phosphate.

**Figure 2:**
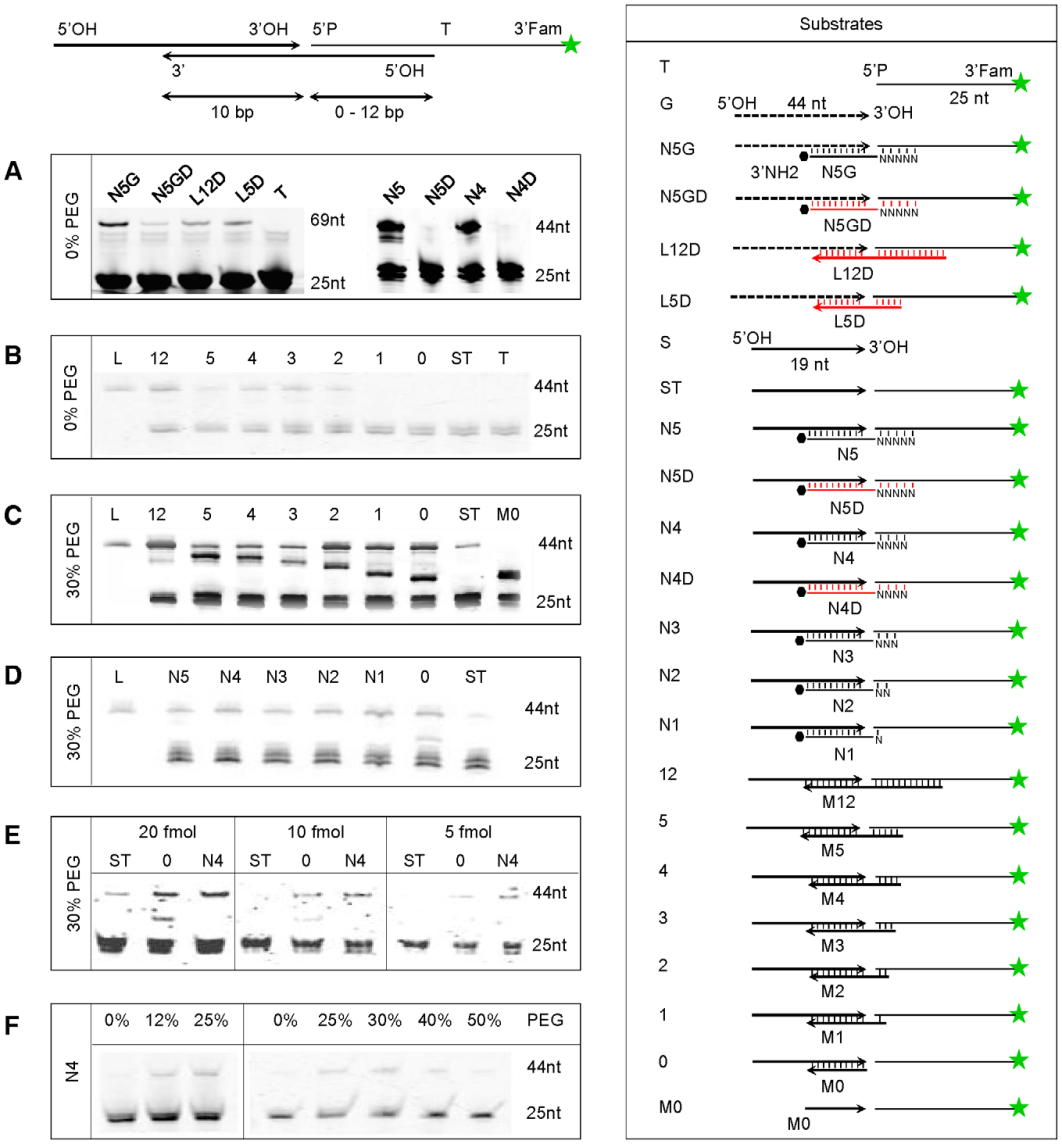
Rnl2 activity on a range of substrates and buffers. Oligonucleotide tagging assays were performed with Rnl2, in various PEG concentrations and the substrates shown on the right panel: the 5′P of the Fam-labelled T oligonucleotide is ligated to the 3′OH of S or G, producing a larger fragment, revealed by urea PAGE and fluorimager scanning. RNA and the mixed RNA-DNA (T) oligonucleotides are shown in black, DNA in red. L: 44 nt RNA-DNA sequence identical to the S-T ligation product. •: 3′ NH2 group. **A**) Rnl2 ligation is best on RNA (N5G, N5 and N4) *vs* DNA-lower-strand substrates. **B**) In 0% PEG, Rnl2 is active on substrates bearing a template strand as short as 12, 5, 4, 3, or 2 nt complementary to T. **C**) In 30% PEG, Rnl2 is also active with the shorter M1 (lane 1) and M0 (lane 0) lower strands and the non-templated ST substrate. **D**) Similar results are observed with random-sequence sticky ends. In (**C**) the ladder of extra bands between 25 and 44 nt corresponds to single-strand ligations between M and T, as confirmed in lane M0; both M12-T (47 nt) and S-T (44-nt) ligation products are not completely resolved on this gel; no such “extra-bands” are observed in (**D**), due to the blocked 3′NH2 of the N5-N1 oligonucleotides. **E**) 5, 10 and 20 T-equivalent fmol of ligation products show best rate for the (N4) templated ligation. **F**) 30% PEG ligation buffer produces best tagging efficiency.

**Figure 3:**
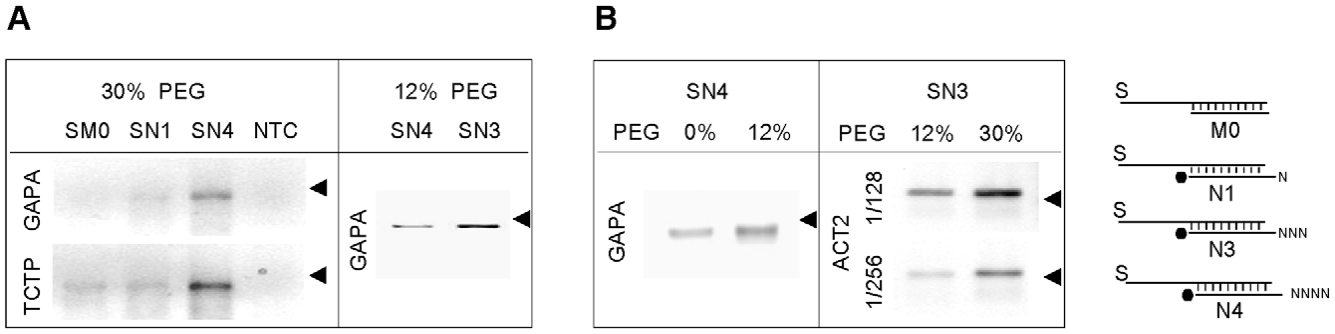
RACE PCR based on various Rnl2-tagging conditions. Total RNAs were ligated to the adapters and in the PEG concentrations shown on top of the gel. Semi-quantitative RACE PCRs were performed on *TCTP*, *GAPA* or *ACT2* transcripts, and analysed on ethydium-bromide stained 4%-agarose gels. Two cDNA dilutions were used for the ACT2 RACE PCR. The bands obtained with the SN3 and SN4 adapters are consistent with the expected RACE products, SN1 and SM0 produced very weak or non-detectable signals. NTC: No template control. Relative cDNA concentration as deduced from QRT-PCR with (ACT2.190 U, ACT2.256L): 1^rst^ panel 1.06 (SM0), 1.44 (SN1), 1 (SN4); 2^nd^ panel 1.33 (SN4), 1 (SN3); 3^rd^ panel 1.08 (PEG 0%) 1 (PEG 12%); 4^rth^ panel 1 (PEG 12%) 1.04 (PEG 30%). ▸ 200 bp. The SN3-adapter and 30% PEG ligation buffer produces best RACE PCR efficiency.

An overview of the proposed cDNA-synthesis strategy is shown in Figure 1. Contrary to previous methods [9], [10], [12], Rnl2 is used to add an oligoribonucleotide to the 5′ end or specifically to the cap site of RNAs. The procedure is based on a 5′ adapter generating a local-double-strand structure with the transcript. The adapter-ligated RNA can then be reverse transcribed with an oligo-dT-primer carrying a second adapter, leading to cDNAs with cloning sites integrated at both ends. This reaction product can be used for producing full-length double-stranded cDNAs or as template in deep sequencing or RACE PCR experiments. The optimum conditions for grafting an adapter on the RNA were developed by using RACE PCR and a straightforward biochemical test. Five RACE PCR assays were designed on well-documented genes in *Arabidopsis*. The transcripts were chosen with increasing expression level (Table S1) ranging from 1 (*eIF4E*) to 88 (*RBCS*) EST counts among 12,273 leaf ESTs in Unigene (http://www.ncbi.nlm.nih.gov/UniGene); the *RBCS* abundant signal was obtained by anchoring the (RBC402L) RACE primer in a region shared by all 4 *RBCS* genes as described previously [12]. The RACE PCRs were carried out in semi-quantitative conditions, on cDNAs normalized by Q-PCR based on *ACT2* (Figure S1). Parallel to this, an oligonucleotide tagging assay using denaturing-gel electrophoresis to detect increases in length of an oligonucleotide upon ligation was derived from previous studies [14] (Figure 2). Based on various oligonucleotide combinations, Rnl2 showed best activity on full RNA adapters (Figure 2A). By contrast comparable nick-joining rates with DNA or RNA template strand were shown in other studies [14], [17]; the discrepancy could be due to the different shorter substrates used in the present study. Lower adapter fragments with gradually shorter sticky ends were assayed in different ligation buffers. While in 0% polyethylene glycol (PEG), 2 bases are sufficient for nick joining by Rnl2 (Figure 2B), in 30% PEG the ligase is also active on one-base sticky ends, blunt-end adapters and single-strand substrates (Figure 2C). Based on homogeneous oligonucleotides this result is confirmed on complex adapters, bearing 1–5 random-base sticky ends (Figure 2D). Although blunt-end or single-strand adapters are sealed, the Rnl2 performed better with sticky end adapters (Figure 2E). This was corroborated by RACE PCRs, showing the SN3 adapter with a 3-random-base sticky end provided best activity on cellular RNAs (Figure 3A). Macromolecular crowding agents are commonly used to improve Dnl or Rnl1 ligations [18]; by RACE PCR (Figure 3B) and oligonucleotide tagging assays (Figure 2F) the best Rnl2 activity is shown in 30% PEG buffer, and by ligating at 25°C then 16°C (Figure S2).

### Rnl2 outperforms Rnl1 and Dnl for tagging the RNA

#### RACE on the cap site of mRNAs

**Figure 4:**
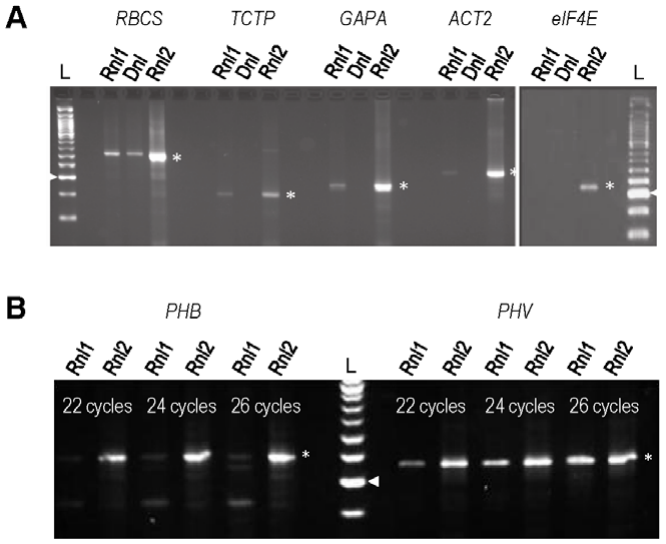
5′ RACE PCR based on RNA tagging by Rnl2, Dnl and Rnl1, towards (A) mRNA cap sites and (B) microRNA-directed cleavage sites. The adapters were ligated (**A**) on dephosphorylated/decapped RNAs or (**B**) untreated RNAs, with the method indicated on each lane. The RACE PCR were performed under semi-quantitative conditions (**A**) on the less and less abundant *RBCS*, *TCTP*, *GAPA*, *ACT2*, *eIF4E* transcripts or (**B**) with 22–26 nested-PCR cycles on the *PHB* and *PHV* cleaved RNAs. Relative cDNA concentrations as deduced from QRT-PCR based on *ACT2* internal primers (**A**) 1.58 (Rnl1), 1.04 (Dnl), 1.00 (Rnl2); (**B**) 1.10 (Rnl1), 1.00 (Rnl2). L: 50 bp ladder. ▸: 200 bp band. *: Bands consistent with the expected major RACE products.

The proposed strategy based on Rnl2 has been compared to the Dnl [12] and the Rnl1 (Oligo Capping) methods [10], [19] with the panel of RACE PCR assays towards the cap site of mRNAs. Complementary DNAs were prepared using the three RNA-tagging protocols, normalized by Q-PCR (Figure S1) and amplified by RACE PCRs (Figure 4A). All five RACE assays showed nice bands on the gel with the RNA Captor (Rnl2) method and less or no visible products with the other two protocols by using the same cDNA amounts and PCR conditions. In particular, Rnl1 tagging produced fainter bands for *RBCS*, *TCTP*, *GAPA and ACT2*, no detectable products for the low-abundant (*eIF4E*) transcript and only the most-sensitive assay (*RBCS*) was positive with the Dnl method (Figure 4A). Similar results were reproduced with different cDNA preparations.

#### RACE on microRNA-directed cleavage site of mRNAs

RACE PCR is widely used to characterizing cleaved RNA products which, just as for non-degraded mRNAs, is hindered by transcript paucity. To show the RNA Captor applies to this topic, a RACE PCR was performed on the two documented miR165/166-targeted *PHB* and *PHV* genes [20] and compared to the Rnl1 RNA-tagging method. Both cleaved RNAs were successfully amplified by RACE, showing the Rnl2 method is convenient for studying degraded transcripts (Figure 4B). Under similar conditions, the Rnl1 method produced fainter bands on the gel and therefore appears less sensitive than the RNA Captor protocol.

**Figure 5:**
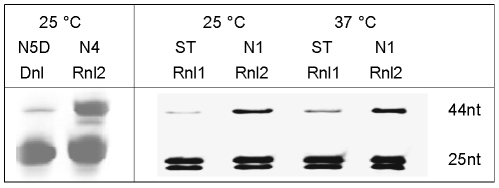
Comparison of Rnl2, Dnl and Rnl1. Oligonucleotide tagging assays were carried out with the ligase, substrate and temperature shown on each lane, in 30% (Dnl and Rnl2) or 25% (Rnl1) PEG ligation buffer. The substrates are described in figure 2. Rnl2 outperforms Dnl and Rnl1 for adding an adapter on the 5′ end of RNAs.

This range of RACE PCRs run on seven transcripts with different expression levels shows that Rnl2 outperforms Rnl1 and Dnl for adding an adapter on the 5′ end of RNAs. The oligonucleotide tagging assay led to the same conclusion (Figure 5).

### Large scale transcript inventories and identification of full-length cDNA for MIR genes

The RNA Captor protocol was used to construct 11 full-length cDNA libraries from grapevine and melon. A preliminary analysis has been run on 53,215 5′ reads corresponding to 11 subsets of 3466 to 9624 clones and 880 to 3280 cDNA clusters from each library. Among the 53,215 clones, 45,359 match to Arabidopsis proteins and 92.6% of these 45,359 are large enough to span the homologous start ATG, suggesting most of the clones are full length. We also looked at the occurrence of micro RNA transcripts among these cDNAs and identified three clones corresponding to primary micro RNAs (pri-miR164, pri-miR168 and pri-miR390) not yet characterized in melon. Primary micro RNAs are transient molecules subjected to maturation, generally under-represented among ESTs; based on EST counts (TAIR, http://www.arabidopsis.org/) and small RNA sequencing data (ASRP, http://asrp.cgrb.oregonstate.edu/db/), these clones correspond to medium to low expressed *MIR* genes in *Arabidopsis*. The RNA Captor protocol is therefore efficient for getting full-length cDNAs corresponding to mRNAs as well as non-protein-coding RNAs.

## Discussion

This study provides an alternative strategy to the difficult issue of transcript characterization. Grafting an oligonucleotide on the 5′-phosphate of RNAs can be carried out by Rnl2 and partially degenerate adapters. The performance of this new method was assessed by seven RACE PCR assays targeted towards the cap or cleavage sites of mRNAs and by constructing 11 full-length cDNA libraries. Preliminary analysis showed 92.6% of potentially full-ORF clones in these collections and three novel melon miRNA primary transcripts corresponding to medium to low expressed *MIR* genes in *Arabidopsis*. Comparing protocols is a tricky question, especially on this topic, the sensitivity of the new RNA-tagging strategy and related methods was assessed by RACE PCRs under rigorous semi-quantitative conditions and by oligonucleotide tagging assay. In this range of experiments, Rnl2 performed significantly better than Rnl1 and Dnl. Obtaining better rates with sticky-end adapters as compared to non-templated ligation is in line with expectation from previous studies [14]. In the present protocol, possible diminution of the overall efficiency owing to the SN3-adapter complexity is compensated by using molar excess of adapters in the tagging reaction (Figure S2B); replacing the short random-nucleotide tail with desoxyribo-inosines led to poor ligation rates (not shown). The Dnl was first proposed to catalyze templated ligation of RNA [12]; this approach was successful in a large-scale transcript inventory of 84,000 clones used for grapevine genome annotation [13]. Rnl2 ligation notably increases the sensitivity of the strategy, in agreement with the comparative analysis by Bullard and Bowater [14] which showed nicked substrates with a 5′-phosphate-RNA strand are most-effectively ligated by this enzyme. With the Rnl1 tagging method, a wide range of 3′OH polynucleotides can be joined to the 5′-phosphate of the RNAs, leading to unwanted side products such as concatemers or RNA circles and loss of the less-abundant transcripts, even though high-molarity adapters should avoid this problem. With the present method, the selectivity offered by templated ligations keeps such a background to a minimum; accordingly no chimeras have been identified in the large-scale sequencing programs we're currently running.

The RNA Captor is a straightforward and accessible protocol; its efficiency is shown on mRNAs and npcRNAs, bearing or not a 5′ cap. This strategy could also apply to other RNA classes such as small RNAs, viral RNA or for studying RNA-protein interactions *in vivo* [21]. A number of deep-sequencing programs engaged in human and various organisms to deciphering the complete catalogue of transcripts [22], [23], transcription start sites [3] or degradation products [2] could benefit from the advantages offered by the present method.

## Materials and Methods

Total RNAs were extracted from 4-week-old *Arabidopsis-thaliana* Col0 aerial-vegetative tissues, as previously described [13]. Polyethylene glycol 8000 (Fluka BioUltra grade ref. 81268) stock solution was prepared by dissolving at 70% w/v in DEPC-treated water, aliquoted and stored at −20°C. To help pipetting, the aliquots were warmed few minutes at 37°C before use.

### Adapters and oligonucleotides

The oligonucleotide sequences are shown in Table S2. RNA oligonucleotides were purchased from Eurogentec and kept as dried aliquots at −80°C. The double-stranded adapters were prepared extemporaneously: Resuspend and mix the upper and lower oligonucleotide at 400 µM each in 10 mM Tris (pH 7.5) and 1 unit/µl of Rnasin Plus. Heat 2 min at 60°C and ramp down to 25°C, at a rate of 3°C per min.

### Oligonucleotide tagging assay

Unless otherwise specified, the ligations were performed with 2 µM of each oligonucleotide, in 10 µl of ligation buffer, supplemented with 1 unit/µl of Rnasin Plus, 0.5 µl of ligase (20 U/µl for Rnl1, 10 U/µl for Rnl2 and 5 U/µl for Dnl), 0%–50% PEG and incubated 3 h at 20°C. The reactions were stopped by 10 µl of formamide buffer (containing 1 mg/ml of bromophenol blue and 10 mM EDTA), 3 min at 95°C and analysed on a 16% polyacrylamide-urea gel (8.3 cm×10 cm, Biorad) in 0.5× TBE (90 mM Tris, 90 mM boric acid, pH 8.3, 2 mM EDTA), at 40 volt/cm for ∼15 min and revealed on a Storm fluorimager (Amersham Biosciences).

### The RNA Captor protocol

To study microRNA-directed cleavage sites and other non-capped RNAs, go straight to the oligonucleotide ligation step.

#### Dephosphorylation

was performed at 50°C, in Tris-EDTA [24]: 2 µg of total RNAs are diluted in H_2_O containing 15 units of Rnasin Plus ribonuclease inhibitor (Promega), heated at 65°C 3 min, equilibrated at 50°C, supplemented with 50 m*M* Tris-HCl (pH 8.5 at 20°C), 0.1 mM EDTA, 15 units of Rnasin Plus and 20 units of Calf-intestinal alkaline phosphatase (New England Biolabs), in a 20 µl-final volume. After 1 h at 50°C, (do not store the reaction on the ice) the volume is completed to 100 µl with H_2_O, phenol: chloroform extracted twice and precipitated with 300 m*M* sodium acetate, 20 µg of ultra-pure glycogen (Invitrogen), and 2.7 volumes of ethanol. The RNA is pelleted at max speed (14 to 20000 g) 10 min, 70%-ethanol washed and air dried few min on the bench.

#### Decapping

The dephosphorylated-RNA pellet is resuspended in H_2_O containing 10 units of Rnasin Plus, heated at 65°C 3 min, equilibrated at 37°C, and digested for 2 h in 15 µl-final volume, with 6 units of tobacco acid pyrophosphatase (Epicentre) in its accompanying buffer supplemented by 10 units of Rnasin Plus. The volume is completed to 100 µl with H_2_O, phenol: chloroform extracted (once) and ethanol precipitated as above.

#### Oligonucleotide ligation

The RNA pellet is resuspended in H_2_O containing 10 units of Rnasin Plus, heated at 65°C 3 min, equilibrated at 25°C and ligated with 40 µM of S:N3 paired adapter, in 10 µl of Rnl2 buffer supplemented with 10 units of Rnasin Plus, 10 units of Rnl2 (NEB, ref M0239S), and 30% PEG. (the PEG stock solution should be warmed at 37°C before pipetting and mixing the ligation reaction carefully by slowly pipetting up and down without making bubbles). The reaction is incubated on a PCR block, 2 h at 25°C, cooled down to 16°C (*e.g.* with a slope of −0.1°C/min) and kept at 16°C for ∼10 h. Adjust to 200 µl with H_2_O and extract with 1 vol. of phenol-chloroform. If they are to be used for constructing a full-length cDNA library, the RNAs are heated at 65°C 3 min and straight purified on ChromaSpin 100-DEPC H_2_O columns (Clontech), this step is not required for RACE PCR experiments. Add 2.5 M ammonium acetate, 20 µg of glycogen, 2.7 volumes of ethanol and centrifuge at max speed 10 min. The pellet is washed with 70% ethanol, and air dried few min on the bench.

#### Single-strand cDNAs

Resuspend the ligated RNA in H_2_O containing 20 units of Rnasin Plus, 2.5 µM of SfidTr primer and 0.5 mM (each) dNTPs, heat at 65°C 3 min, equilibrate at 50°C and complete to 20 µl with 20 units of Rnasin Plus, 5 m*M* DTT, 50 m*M* Tris-HCl (pH 8.3), 75 m*M* KCl, 3 m*M* MgCl_2_, and 200 units of M-MLV Superscript III (Invitrogen). Incubate at 50°C 35 min then 55°C 15 min, stop the reaction at 70°C 15 min and digest with 1 unit of RnaseH (Invitrogen) at 37°C 20 min. Adjust to 200 µl with H_2_O and extract with 1 volume of phenol-chloroform. If they are to be used for constructing a full-length cDNA library, the cDNAs are heated at 65°C 2 min and straight loaded on ChromaSpin 100 columns (Clontech), this purification is not required for RACE PCR experiments. Precipitate with 2.5 M ammonium acetate, 20 µg of glycogen, 2.2 volumes of ethanol and centrifuge at max speed 10 min. The pellet is washed with 70% ethanol, and air dried few min on the bench. ss cDNAs can be stored in 70% ethanol or as dried pellet.

#### RACE PCRs

Although single-strand cDNAs can be used as template in RACE PCRs, best results were obtained with double-strand cDNAs produced in the following highly-stringent conditions. Resuspend the first-strand reaction in 20 µl-final volume of ExTaq buffer containing 2 mM of MgCl_2_, 200 µM of each dNTPs, 0.1 µM of each primer SD and SfidTp, heat at 94°C 20 sec, cool to 72°C, add 1 unit of (Takara) ExTaq, proceed with 72°C 8 min, 94°C 20 sec, 70°C 8 min, 94°C 20 sec, 69°C 8 min, 94°C 20 sec, 68°C 8 min. Do a phenol-chloroform extraction in 200 µl, add 2.5 M ammonium acetate, 20 µg of glycogen, 2 volumes of ethanol and centrifuge at max speed 10 min. Wash the pellet with 70% ethanol, air dry few min on the bench and resuspend in 20 µl of Tris 10 mM (pH 8) EDTA 0.1 mM. For the present study, 1 µl of cDNAs (or dilutions) were amplified with 1 unit of Taq polymerase (NEB) in 15 µl of buffer containing 200 µ*M* of each dNTPs, 0.3 µ*M* of gene-specific primer and 0.9 µM of SD adapter primer. Hot starts were performed by adding the primers at the first hybridization step. Thermal cycling: 94°C 20 sec then [94°C 20 sec, 58°C 10 sec, 72°C 40 sec] 35 cycles (for *RBCS*, *TCTP*, *GAPA*, *ACT2*) or 40 cycles (for *eIF4E*). The miR165/166-targeted cleavage sites of *PHB* and *PHV* genes were amplified using the gene-specific primers described by Mallory *et al* [20]. For these less-abundant transcripts, I performed highly-specific hemi-nested PCRs by avoiding cooling down the reaction below the hybridization temperature between the first and the second PCR. Start the first PCR with (PHB1 or PHV1, SD), as above specified with a thermal cycling program of 94°C 20 sec and 52 cycles of [94°C 20 sec, 60°C 20 sec, 72°C 20 sec]. At the 26^th^ cycle, transfer 1 µl of the reaction to the pre-heated second-PCR mix, containing PHB2 or PHV2 and SD primers, and carry on the cycling to a total number of 48, 50 or 52 cycles. The expected major RACE PCR fragments are: 320 bp with RBC402L, ∼130 bp with TCTP142L; ∼180 bp with GAPA143L; 220 bp with ACT2.256L; 250 bp with eIF4E215L; 228 bp with PHB2 and 231 bp with PHV2.

#### Construction of full-length cDNA library

for the grapevine and the melon transcript inventories, the libraries were constructed in suboptimal conditions, with small amount of RNA, tissues infected by pathogens or with high-sugar content. Depending on available starting material, the inserts were produced from 4 µg of total RNA, to 2 µg of total RNA enriched with 200 ng of poly(A)^+^ RNA, to 8 µg of poly(A)^+^ RNA. The first-strand cDNAs were resuspended in 85 µl of 1× ExTaq buffer containing 2 mM MgCl_2_, 200 µM each dNTP, 0.2 µM each primer (SD, SfidTp) and 7 units of (Takara) ExTaq, added at the first annealing step (72°C) and amplified with the following thermal cycling: 94°C 20 sec, 72°C 4 min, 94°C 20 sec, 70°C 4 min, 94°C 20 sec, 69°C 4 min and 6–15 cycles of [94°C 20 sec, 68°C 4 min]. The PCR product was extracted with 1 volume of phenol chloroform, purified on a Chromaspin 100 column (Clontech) and digested in 150 µl with 20 units of *Sfi*1 (NEB) 2 h at 50°C. Extract by 1 volume of phenol chloroform, ethanol precipitate, 70%-ethanol wash, air-dry 2 min and resuspend in 7 µl of Tris 10 mM (pH 8), EDTA 1 mM. The inserts above 1 kbp were fractionated on 1.5%-agarose gel, electroeluted, ethanol precipitated and resuspended in 10 µl Tris HCl pH 7.6 10 m*M*. Around 20 to 100 ng of digested cDNAs were ligated with 4 ng of *Sfi*1-digested pLUD vector [13] with 0.5 µl of T4 DNA ligase [5 units/µl] (Invitrogen) in 15 µl of supplied buffer, over night at 16°C. The ligations were spin dialysed and electroporated into *E. coli* DH10B T1^r^ (F^−^ *mcr*A Δ(*mrr-hsd*RMS-*mcr*BC) Φ80*lac*ZΔM15 Δ*lac*X74 *deo*R *rec*A1 *end*A1 *ara*Δ139 Δ (*ara,leu*)7697 *gal*U *gal*K λ^−^ *rps*L (Str^R^) *nup*G *ton*A), with a Life Technologies electroporator, following the manufacturer's conditions and plated on LB agar plates supplemented with carbenicillin (50 µg/ml), 5-bromo-4-chloro-3-indolyl-β-D-galactoside (20 µg/ml) and isopropylthio-β-D-galactoside (20 µg/ml). The ligations were titrated between 4 and 40 millions CFU per µg of insert, with a 10^−4^ vector background. Overall between 0.2 and 4.5 millions clones were obtained for each library.

### Comparison of the RNA-tagging methods based on Rnl2, Rnl1 and Dnl

The RNA taggings were carried out in parallel, using the same S oligoribonucleotide aliquot with 2 µg equivalent of the same dephosphorylated and decapped RNA preparation for the RACE PCR towards the cap sites and 2 µg of the same untreated total RNA aliquot for amplifying the cleavage sites. Rnl1 ligations on the RNA 5′end with the S oligoribonucleotide were performed as recommended by Suzuki and Sugano [19]; other assays carried out in a 20 µl-final volume (instead of 100 µl) with 1 µM of S adapter (instead of 0.42 µM), the New-England-Biolabs Rnl1 (instead of Takara Rnl1) and overnight incubations produced similar ligation rates. Dnl-based RNA ligations were performed as described previously [12] but for using 10 µM of annealed [S : N5D] adapter and 30%-PEG buffer. After oligonucleotide ligation, all RNAs were processed identically.

### Q-RT PCRs

The cDNAs were normalized by quantitative-RT PCR with ACT2-internal primers (see Figure S1). The linearity of amplification with (ACT2.190U, ACT2.256L) was evaluated using triplicate serial dilutions (1∶1, 1∶2, 1∶4, 1∶8, 1∶16 and 1∶32) of 1 µl of single-strand cDNA template (*i.e.* 100 ng equivalent of starting total RNA) on an ABI PRISM 7900 system (Applied Biosystems), with 0.3 µM of each primers in 10 µl of 1× MESA-Green Q-PCR mix (Eurogentec) and a thermal cycling of 50°C 2 min, 95°C 10 min and 38 cycles [95°C 15 sec, 58°C 1 min]. The resulting standard plot (Cq versus cDNA concentration input) was characterized by a slope = −3.35079 and a correlation coefficient R^2^ = 0.99846. A primer efficiency, E = 98.8%, was calculated for this ACT2 primer pair, according to the equation E = (10^−1/slope^−1)×100. The relative concentration of the various cDNA preparations was calculated with the 2^−ΔCt^ method; the Q-RT PCRs were run with 1 µl of cDNA or 10-fold dilutions with the above conditions. The specificity of the Q-RT PCR products were routinely confirmed on 3%-agarose gel, expected size is 87 bp from cDNA and 587 bp from genomic DNA.

### Sequence analyses and accession numbers

BLAST searches [25] were performed against the *Arabidopsis* proteome (Flagdb, [26]) or miRBase (http://www.mirbase.org/). The three new melon pri-miRNA sequences were deposited in miRBase under the following accession number MI0018163 (cme-MIR164), MI0018164 (cme-MIR390) and MI0018177 (cme-MIR168).

## Supporting Information

Figure S1 **Normalization of the cDNAs.** QRT-PCR based on *ACT2* of cDNAs produced by RNA tagging using the Rnl2, Dnl or Rnl1 method. Separated by a 500 bp intron on genomic DNA, the ACT2.190 U and ACT2.256L primers favor PCR amplification of the shortest cDNA fragment. The specificity of the Q-PCR reaction is confirmed by agarose-gel analysis, showing a single band at the expected size (87 bp). As shown on the Standard Plot, the Ct values are completely proportional to the amount of cDNAs used in the Q PCR assay. The amplification plot and accompanying histogram show the relative concentration of the cDNAs used in Figure 2a. NTC: no template control.
(TIF)

Figure S2 **Oligonucleotide tagging assays with Rnl2 under various conditions.** Ligations were performed with Rnl2, on the indicated substrates, in 10 µl of 30% PEG ligation buffer, in the following specific conditions: **A**) Ligations with 2 µM of each oligonucleotide and a range of 0.3 to 20 units of ligase, for 3 h at 25°C. 10 fmol equivalent of T oligonucleotide were loaded per well. Between 2.5 and 10 Rnl2 units produced best ligation rates. **B**) Ligations with 1 unit of ligase, 2 µM of T oligonucleotide and increasing amount (0.02–100 µM) of the N4-adapter, as shown above the lanes. One hundred fmol equivalent of T oligonucleotide were loaded per well. The tagging efficiency reaches a plateau from 2 to 100 µM of adapter in the tagging reaction, without obvious inhibition at the highest concentrations. Any possible reduction of the overall ligation efficiency expected from the use of a complex adapter (4^3^ = 64 combinations for the retained N3-adapter) can therefore be compensated by using molar excess of adapters in the tagging reaction. **C**) Ligations with 5 units of ligase and 2 µM of each oligonucleotide were incubated at 37 or 25°C for 1 h. For each reaction 200, 50 and 12 fmol equivalent of T oligonucleotide were loaded. No ligation-rate differences are shown between both conditions, confirming the PEG ligation can be performed at low temperature, as recommended for the other two T4 ligases. **D**) Ligations with 5 units of ligase and 2 µM of each oligonucleotide were incubated for 1 h at 25°C, followed or not by a second incubation of 7 h at 16°C. Eighty fmol equivalent of T oligonucleotide were loaded per well. Slightly improved ligation rates were obtained by keeping the reactions few additional hours at 16°C. L: 44 nt oligonucleotide control.
(TIF)

Table S1 **Expression level of the genes used in the RACE PCR assays.** EST counts of various *Arabidopsis* genes among 12,273 leaf ESTs in Unigene (http://www.ncbi.nlm.nih.gov/UniGene; 01/25/2010 release). Libraries known to be normalized, subtracted or otherwise biased were not included in this profile by Unigene. In particular in this pool of ESTs, the RBC402L primer matches (53+3+7+25 = ) 88 ESTs from all four *RBCS* genes.
(TIF)

Table S2 **Oligonucleotides.** The adapters and primers retained for the RNA Captor protocol are in the upper part of the table. r: RNA/d: DNA backbone; T and L are mixed RNA/DNA oligonucleotides. rN: A, G, C or U; dN: dA, dG, dC or dT; dV: dA, dG or dC. 5′P: 5′ phosphate.
(TIF)
