# Supplementary figures and images for: RNA Captor: A Tool for RNA Characterization
Source: PLoS One. 2011 Apr 13;6(4):e18445. doi: 10.1371/journal.pone.0018445 (PMC3076428; doi:10.1371/journal.pone.0018445)

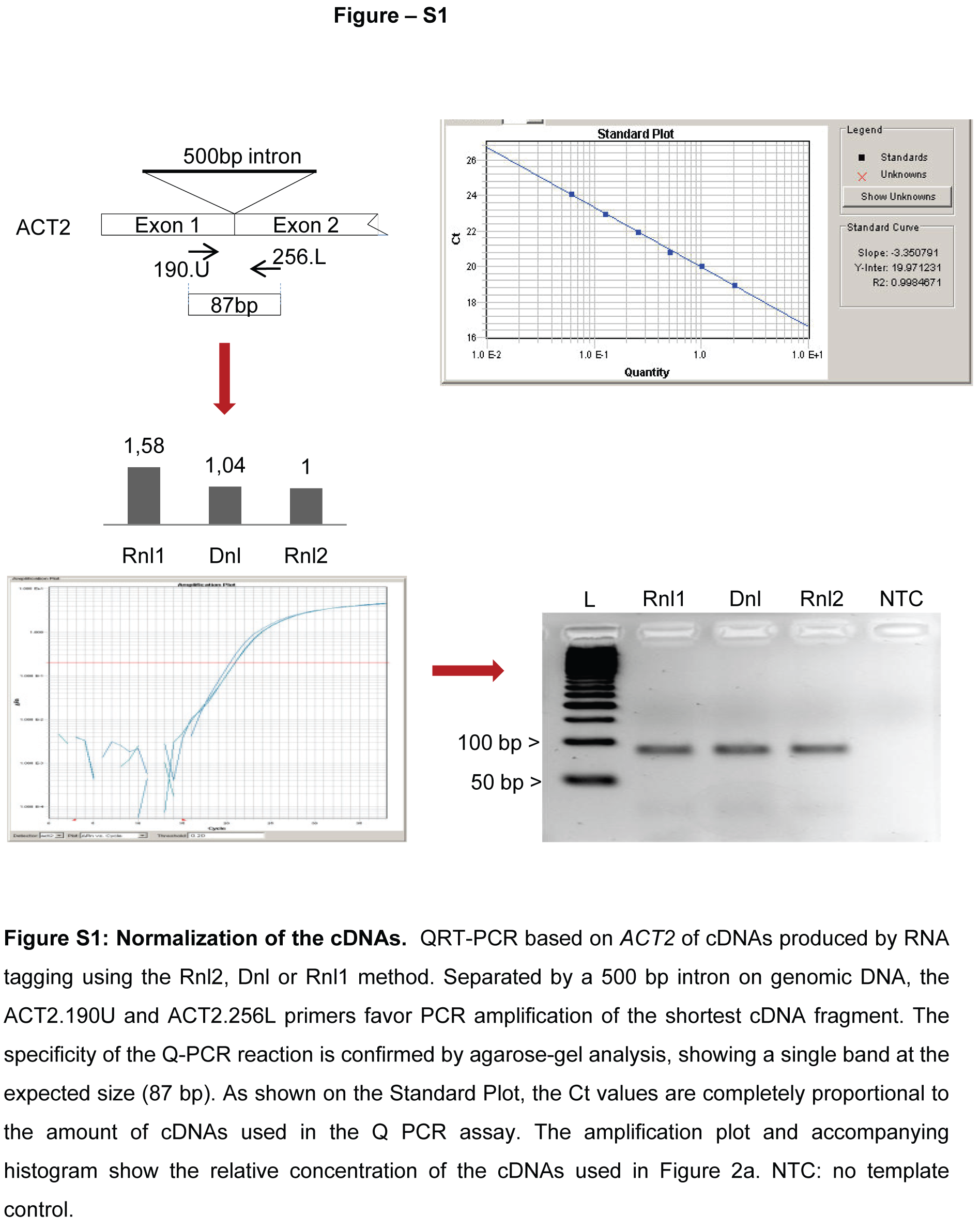

Supplement: Figure S1 — Normalization of the cDNAs. QRT-PCR based on ACT2 of cDNAs produced by RNA tagging using the Rnl2, Dnl or Rnl1 method. Separated by a 500 bp intron on genomic DNA, the ACT2.190 U and ACT2.256L primers favor PCR amplification of the shortest cDNA fragment. The specificity of the Q-PCR reaction is confirmed by agarose-gel analysis, showing a single band at the expected size (87 bp). As shown on the Standard Plot, the Ct values are completely proportional to the amount of cDNAs used in the Q PCR assay. The amplification plot and accompanying histogram show the relative concentration of the cDNAs used in Figure 2a. NTC: no template control. (TIF) [file pone.0018445.s001.tif]

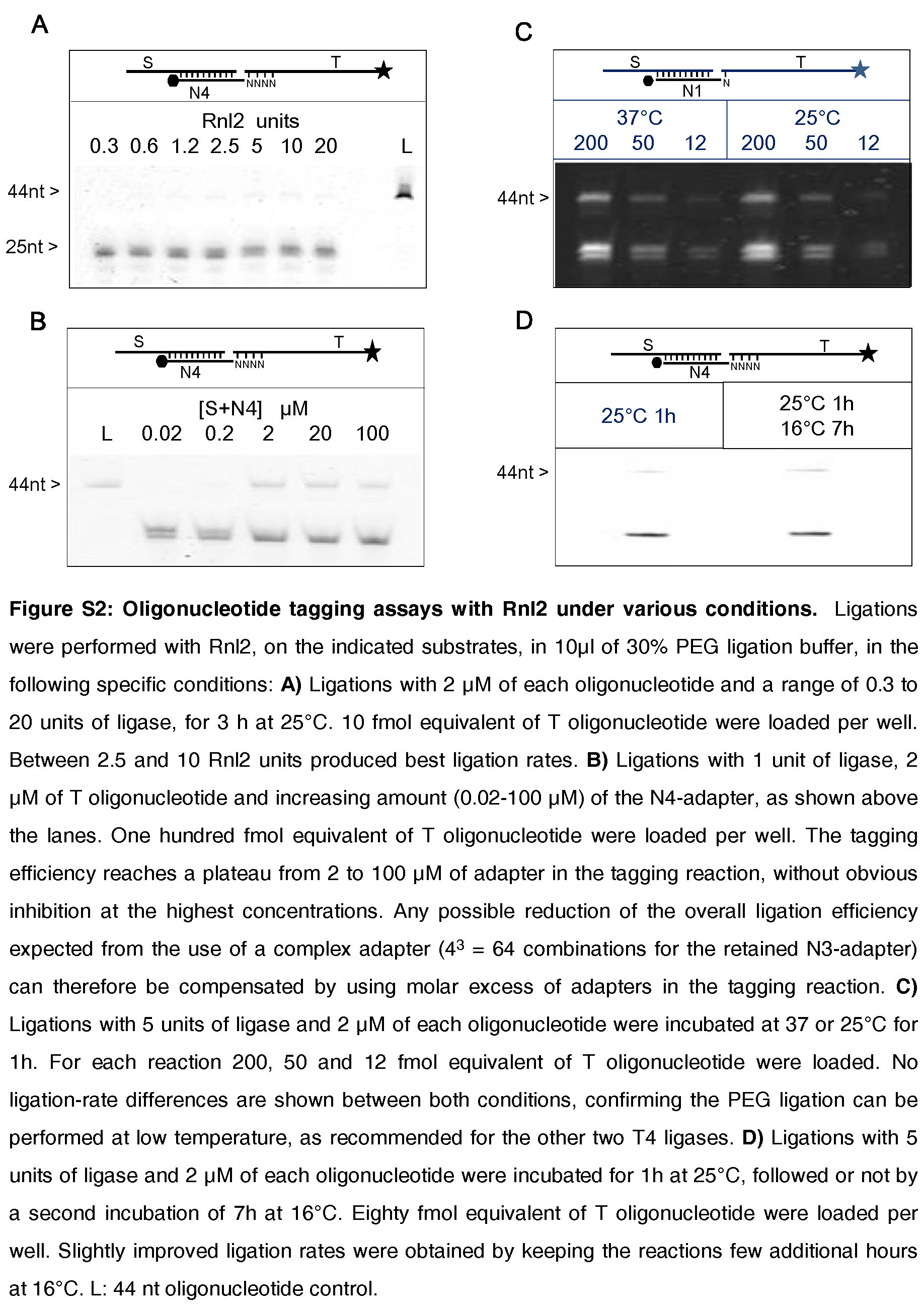

Supplement: Figure S2 — Oligonucleotide tagging assays with Rnl2 under various conditions. Ligations were performed with Rnl2, on the indicated substrates, in 10 µl of 30% PEG ligation buffer, in the following specific conditions: A) Ligations with 2 µM of each oligonucleotide and a range of 0.3 to 20 units of ligase, for 3 h at 25°C. 10 fmol equivalent of T oligonucleotide were loaded per well. Between 2.5 and 10 Rnl2 units produced best ligation rates. B) Ligations with 1 unit of ligase, 2 µM of T oligonucleotide and increasing amount (0.02–100 µM) of the N4-adapter, as shown above the lanes. One hundred fmol equivalent of T oligonucleotide were loaded per well. The tagging efficiency reaches a plateau from 2 to 100 µM of adapter in the tagging reaction, without obvious inhibition at the highest concentrations. Any possible reduction of the overall ligation efficiency expected from the use of a complex adapter (43 = 64 combinations for the retained N3-adapter) can therefore be compensated by using molar excess of adapters in the tagging reaction. C) Ligations with 5 units of ligase and 2 µM of each oligonucleotide were incubated at 37 or 25°C for 1 h. For each reaction 200, 50 and 12 fmol equivalent of T oligonucleotide were loaded. No ligation-rate differences are shown between both conditions, confirming the PEG ligation can be performed at low temperature, as recommended for the other two T4 ligases. D) Ligations with 5 units of ligase and 2 µM of each oligonucleotide were incubated for 1 h at 25°C, followed or not by a second incubation of 7 h at 16°C. Eighty fmol equivalent of T oligonucleotide were loaded per well. Slightly improved ligation rates were obtained by keeping the reactions few additional hours at 16°C. L: 44 nt oligonucleotide control. (TIF) [file pone.0018445.s002.tif]

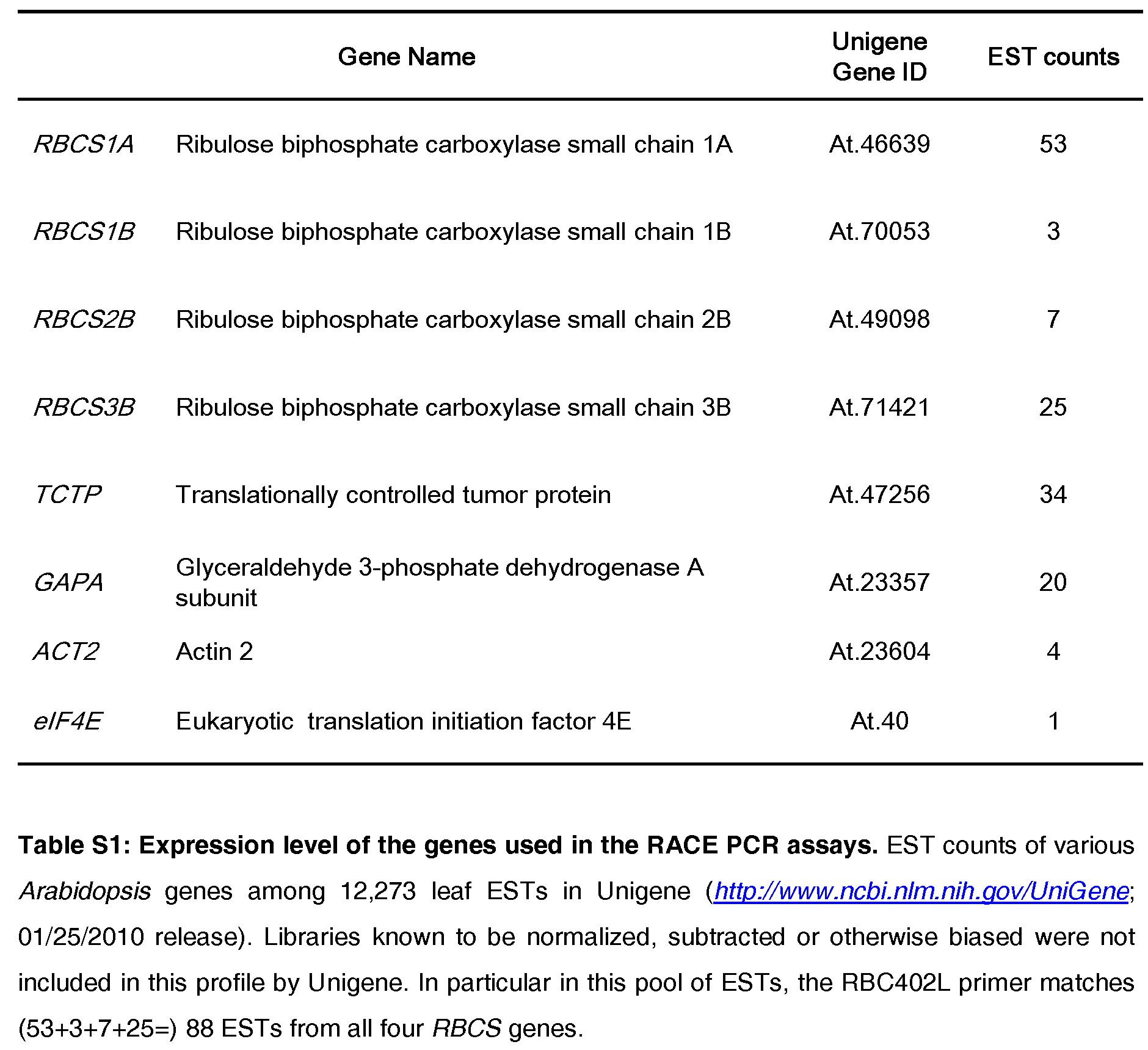

Supplement: Table S1 — Expression level of the genes used in the RACE PCR assays. EST counts of various Arabidopsis genes among 12,273 leaf ESTs in Unigene (http://www.ncbi.nlm.nih.gov/UniGene; 01/25/2010 release). Libraries known to be normalized, subtracted or otherwise biased were not included in this profile by Unigene. In particular in this pool of ESTs, the RBC402L primer matches (53+3+7+25 = ) 88 ESTs from all four RBCS genes. (TIF) [file pone.0018445.s003.tif]

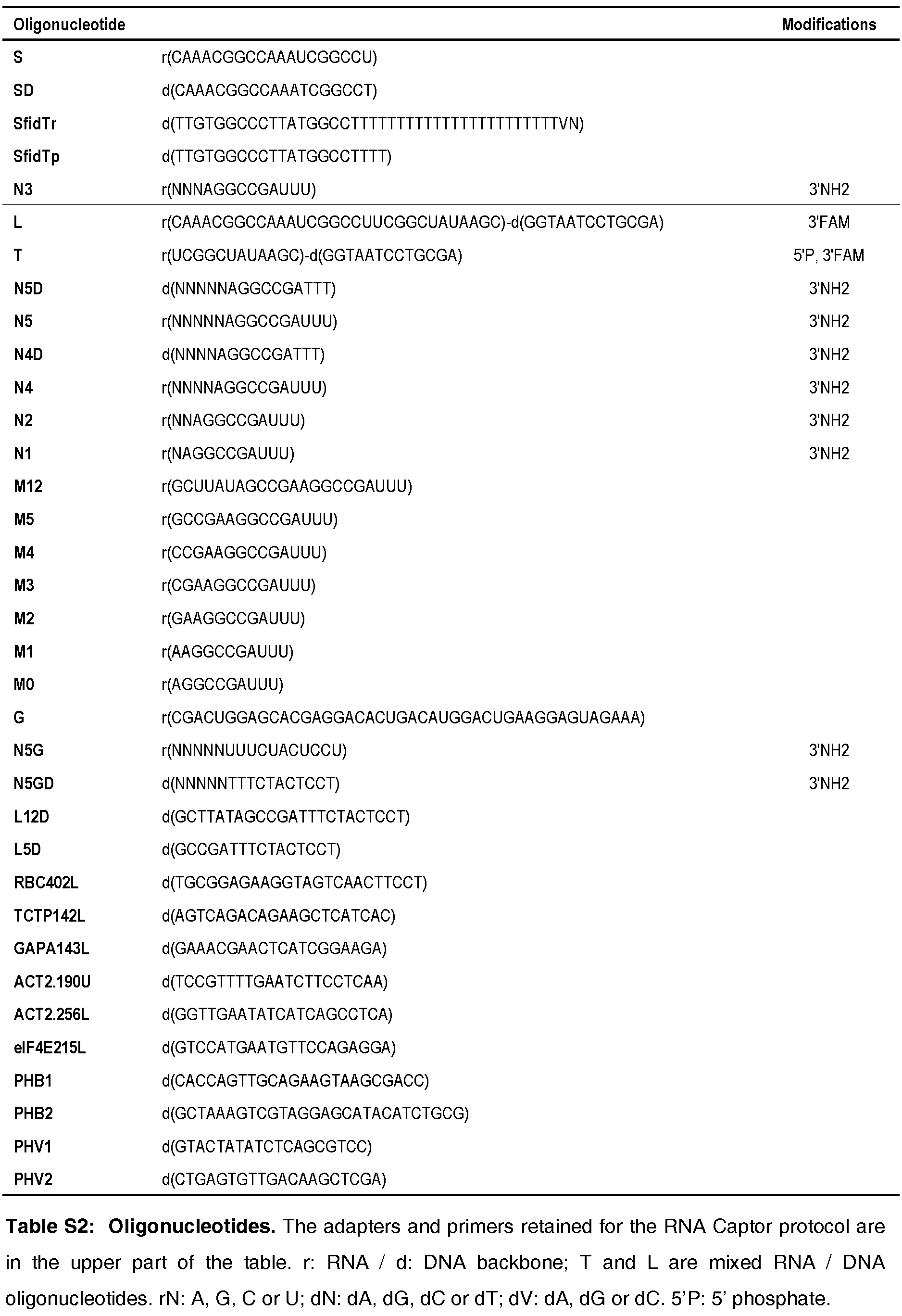

Supplement: Table S2 — Oligonucleotides. The adapters and primers retained for the RNA Captor protocol are in the upper part of the table. r: RNA/d: DNA backbone; T and L are mixed RNA/DNA oligonucleotides. rN: A, G, C or U; dN: dA, dG, dC or dT; dV: dA, dG or dC. 5′P: 5′ phosphate. (TIF) [file pone.0018445.s004.tif]
